# Supplementary material for: Distribution of an analgesic palmitoylethanolamide and other N-acylethanolamines in human placental membranes
Source: PLoS One. 2023 Jan 13;18(1):e0279863. doi: 10.1371/journal.pone.0279863 (PMC9838831; doi:10.1371/journal.pone.0279863)
Supplement: S2 Table — The descriptive statistics for each data set was calculated using R package. Only data with a P-value of ≤ 0.05 were considered statistically significant. (DOCX) [file pone.0279863.s002.docx]

**Supporting_S2 Table:**

**Supporting_S2 Table: Concentrations of PEA, OEA, and AEA (ng/g; ng/ml for US) in various placental specimens - original data and statistics (AV, average; SD, standard deviation, values of triplicates). The descriptive statistics for each data set was calculated using R package. Only data with a p-value of ≤ 0.05 were considered statistically significant.**

**PEA**

| G | Gender of newborn child | | |
| --- | --- | --- | --- |
| M | Male |  |  |
| F | Female |  |  |

**OEA**

**AEA**
