# Supplementary material for: Distribution of an analgesic palmitoylethanolamide and other N-acylethanolamines in human placental membranes
Source: PLoS One. 2023 Jan 13;18(1):e0279863. doi: 10.1371/journal.pone.0279863 (PMC9838831; doi:10.1371/journal.pone.0279863)
Supplement: S3 Table — AV, average; SD, standard deviation; PL, placenta. (DOCX) [file pone.0279863.s003.docx]

**Supporting_S3 Table:**
