# Supplementary material for: Distribution of an analgesic palmitoylethanolamide and other N-acylethanolamines in human placental membranes
Source: PLoS One. 2023 Jan 13;18(1):e0279863. doi: 10.1371/journal.pone.0279863 (PMC9838831; doi:10.1371/journal.pone.0279863)
Supplement: Attachment — Submitted filename: PLOS ONE_PEA_Response to Reviewers.docx [file pone.0279863.s004.docx]

We duly considered all comments and suggestions of the reviewers, and we made the appropriate corrections and amendments. We appreciated all reviewers' suggestions, and we believe that they contributed to the improvement of the manuscript. All changes that we made in the manuscript are highlighted in yellow.

To the comments of the editor:

**1. For best readability, Please convert the table 2 and the supplemental table 3 in graphs (for example histograms). Include the figures from supplemental table 3 as new figures of the paper, because these data may be useful and inherent.**

**Please respond accurately to each point of reviewer 1.**

Table 2 and supplemental Table 3 are now presented as histograms (Fig.1 and Fig. 2, respectively) as editor suggested.

**Reviewers' comments:**

**Reviewer #1:**

**1.The submitted study offers a direct measurement of N-acylethanolamines (natural pain killers) in different part of full-term human placentae. The authors performed ultra-HPLC in tandem with MS to determine and quantify bioactive lipids with analgesic activity in human placental tissues. The results are of particular interest to current and future clinical activities. However, the study is quite preliminary and limited in the quality and quantity of measurement. The analgesic effect offered by human placenta, and amnion membrane in particular, has been previously described and recently tested in monocentric trial (Mohseni F et al., Crescent Journal of Medical and Biological Sciences 2018). The study performed by Svobodova and co-authors should be considered as short communication since the authors limitedly measured presence of three selected NAE compounds (PEA, OEA and AEA) in 7 specimens.**

The analgesic effect of placenta was repeatedly investigated and evaluated (PMID: 32832040, 32410269, 30559055, study by Mosheni et al., and also in our study: PMID: 34791774), on the other hand the mechanism of this analgesic effect was not related to effector molecules (see Introduction, second paragraph). We believe that triplicates from 7 placentas provide a sufficiently representative set in the terms of study reproducibility. This is evidenced by practically identical results (averages) from the area of AM1 and AM2 and PL1 and PL2, respectively.

**2. The effects of such lipid mediators have been proposed and hypothesized (by the authors and others) related to their action on cannabinoid or PPAR receptors. Unfortunately, none of such hypotheses and pathways have been further elucidated or investigated.**
Yes, this is true, but regarding human study, we have no idea, how to confirm clinically that NAEs are responsible for analgesic action documented after the application of AM to patient’s wound. We would like to mention, that we also detected PPAR receptor in AM, but these results will be included in the following study. Beside that, the presence of PPAR receptor in the amniotic membrane does not indicate directly that reaction between PEA and PPAR is responsible for analgesic effect of the amniotic membrane.

**3. Furthermore, experimental settings is sometime confused and poorly described:
What’s the difference between AM and ACM?**

It is generally known that the difference between AM and ACM lies in the structure and biological properties. The following text has been added to the Introduction section:
The AM is about 0.02-0.05 mm mm thick the innermost layer of the placenta, and is composed of a single epithelial layer, a thick basement membrane and an avascular stroma. The ACM forms the outermost layer of the amniotic sac that surrounds the fetus during pregnancy, while the innermost layer of this sac is the AM. The presence of stem cells, biological active compounds and thick stroma make both tissues an optimal scaffold for wound healing.

**4. Please offer better description (and supportive images) for the “different areas of placental disc” (at page 7 described as “the area near the umbilical cord (AM1, PL1) and the area in the periphery of the placenta”). Are the authors referring to the main placenta body (containing decidua and villi) and reflected amnion/chorion surrounding the baby?**

The description of the areas the tissue was obtained from, was described in the detail, proper references were also added to M and M section:

As concentrations of some proteins are not homogeneously distributed within placenta and AM [65], we collected samples from two different areas of placental disc (PL): from the area near the umbilical cord described as central amniotic membrane or placenta (AM1, PL1) and the area at the edge of placental disc (periphery of the placenta, AM2, PL2) (Centurione 2018, PMID: 29562779, Weidinger 2021, 33520964). The ACM (amniochorionic membrane) consisting reflected amnion from mid zone of chorionic leave was used for experiments (Centurione 2018, PMID: 29562779). The particular locations of AM/ACM are well schematized in a recent paper, which has been included as one of the references (Weidinger 2021, 33520964).

**5. Even the measurements on umbilical cord extracts and UC blood are quite poor and deserve additional profiling and characterization. What about amniotic fluid? why the authors did not include such additional tissue (currently used in clinical setting)?**

The selection of the monitored tissues was based not only on the anatomical point of view (placenta derivatives), but also on the potential importance of the lipids in the tissue. This means that we have focused mainly on tissues that are used clinically for wound treatment and have a strong analgesic effect, i.e. AM and ACM. The levels of amniotic fluid were already assessed (see ref. Discussion, third paragraph). In our experiments, the amniotic fluid was not examined as we could focus just on tissue which is available as a surplus tissue at standard caesarean section. Additionally, the information that UC is covered by AM was included in M and M section.

**6. Please motivate the comparison with selected fetal tissue (Vernix caseosa).**

Vernix caseosa (VC) is a complex biofilm composed of water in hydrated corneocytes (80%), surrounded by a matrix of lipids (10%) and proteins (10%) (PMID: 18489296, 19881987). The lipid fraction is extremely rich and not fully characterized yet, despite the efforts of numerous researches (PMID: 16628195, 28576934). Our motivations were as follows: The discovery of high concentrations of compounds could indicate the analgesic effects of vernix caseosa. Concentration levels of NAEs can also indicate, where NAEs are biosynthesized. (If they are transported to the placenta from the fetal skin.) As it is well known that vernix caseosa protects newborn baby and has some protective roles during fetal development and for first hours after birth, we were interested if in our study detected NAEs are present in this substance. The information about high level of lipids in VC was added to Discussion section.

**7. Introduction is particularly long and contains several information poorly or no pertinent with the current study. Discussion (above all first page) consists in repetition of the information detailed in the Introduction (please revise and erase).**

The Introduction and Discussion were shortened in line with the reviewer's requirements, the other parts required by reviewers (see above) were included.

**8. In Material and Method section, Statistic paragraph, the authors referenced “descriptive statistics for each data set was calculated using R package” (ref. nr.67). Please motivate. In the same paragraph, the authors stated as “The significance of differences in NAEs’ concentrations between the fresh (control) and decontaminated samples of AM/ACM and placenta (PL1, PL2) were tested by Wilcoxon test”. Please motivate both the use of Wilcoxon and the comparison between selected groups (instead of ANOVA or similar).**
Rationales (reasoning) for using Wilcoxon test:

In this study we performed only pair-wise comparison of data sets (AM fresh vs AM dried or PL1 vs PL2). Therefore, multiparametric approaches as ANOVA analysis are not appropriate. As the data sets are quite restricted the normality of the distribution is not possible to confirm (therefore the classical student t-test can be objected) and thus the Wilcoxon test (paired) is the most appropriate to obtain the information about the statistical significances of the differences of the data sets.

**Table 2 and Figure 1 can be combined.**
Table 2 and Figure 1 were combined as suggested by reviewer.

**Discussion is quite extended, and several speculations are included, considering the extremely limited amount of presented data**

Discussion was shortened complying with the reviewer's requirements.

**Reviewer #2: I found this Research paper well written.
Experimental plan is well performed.
Discussion is clear.
Materials and methods section ensures reproducibility.
As limitation, the specific topic could be interested only for very specific readers.**
