# Supplementary material for: Distribution of an analgesic palmitoylethanolamide and other N-acylethanolamines in human placental membranes
Source: PLoS One. 2023 Jan 13;18(1):e0279863. doi: 10.1371/journal.pone.0279863 (PMC9838831; doi:10.1371/journal.pone.0279863)
Supplement: Attachment — Submitted filename: PONE-D-21-41101R3_Response to Reviewers.docx [file pone.0279863.s006.docx]

Prague, November 27, 2022

PONE-D-21-41101R3
Distribution of an analgesic palmitoylethanolamide and other N-acylethanolamines in human placental membranes
PLOS ONE

**Reviewer #1:**

**Please, consider, if possible, to add new data on PPAR-alpha.**

**Our reply:**

We are working on the detection of PPARα receptor in amniotic membrane (AM), but since we want to publish data on PEA concentrations in AM preserved and stored under different conditions (cryopreserved, lyophilized, dry), we plan to accompany these results by images with immunohistochemically detected PPARα for each of technique used.

For this reason, we do not think it would be appropriate to show just incomplete data on PPARα in this paper. Additionally, PPARα is likely important for the analgesic effect particularly in the skin, which is target tissue of AM application. Its expression there has been already detected. Please see Results l. 328 – 332.

As an internal information, I include an image from the detection of PPARα in AM to this answer.

Immunohistochemistry for PARPα (green) in AM section. The nuclei are counterstained with propidium iodide (red).

It should be noted that the supplemental PPARα studies are quite demanding in time and resources and inclusion of even only partial results would require an extensive modification of some parts of the manuscript - Materials and Methods, Results, Discussion.

On the other hand, should you insist on adding PPARα data we would accept it.
